# Effect modification of air pollution on Urinary 8-Hydroxy-2'-Deoxyguanosine by genotypes: an application of the multiple testing procedure to identify significant SNP interactions

**DOI:** 10.1186/1476-069X-9-78

**Published:** 2010-12-07

**Authors:** Cizao Ren, Pantel S Vokonas, Helen Suh, Shona Fang, David C Christiani, Joel Schwartz

**Affiliations:** 1 Exposure, Epidemiology, and Risk Program, Department of Environmental Health, Harvard School of Public Health. Boston, MA. USA; 2 VA Normative Aging Study, Veterans Affairs Boston Healthcare System and the Department of Medicine, Boston University School of Medicine, Boston, MA, USA; 3 Environmental and Occupational Medicine and Epidemiology Program, Department of Environmental Health, Harvard School of Public Health, Boston, MA, USA

## Abstract

**Background:**

Air pollution is associated with adverse human health, but mechanisms through which pollution exerts effects remain to be clarified. One suggested pathway is that pollution causes oxidative stress. If so, oxidative stress-related genotypes may modify the oxidative response defenses to pollution exposure.

**Methods:**

We explored the potential pathway by examining whether an array of oxidative stress-related genes (twenty single nucleotide polymorphisms, SNPs in nine genes) modified associations of pollutants (organic carbon (OC), ozone and sulfate) with urinary 8-hydroxy-2-deoxygunosine (8-OHdG), a biomarker of oxidative stress among the 320 aging men. We used a Multiple Testing Procedure in R modified by our team to identify the significance of the candidate genes adjusting for *a priori *covariates.

**Results:**

We found that glutathione S-tranferase P1 (GSTP1, rs1799811), M1 and catalase (rs2284367) and group-specific component (GC, rs2282679, rs1155563) significantly or marginally significantly modified effects of OC and/or sulfate with larger effects among those carrying the wild type of GSTP1, catalase, non-wild type of *GC *and the non-null of GSTM1.

**Conclusions:**

Polymorphisms of oxidative stress-related genes modified effects of OC and/or sulfate on 8-OHdG, suggesting that effects of OC or sulfate on 8-OHdG and other endpoints may be through the oxidative stress pathway.

## Background

Many studies have shown that ambient pollution is consistently associated with adverse health outcomes [1-6], but mechanisms accountable for these associations have not been fully elucidated. Suggested biological mechanisms linking air pollution and cardiovascular diseases include direct effect on the myocardium, disturbance of the cardiac autonomic nervous system, pulmonary and systematic oxidative stress and inflammatory response that triggers endothelial dysfunction, atherosclerosis and coagulation/thrombosis [7]. Understanding relative roles of such potential is a priority of recent air pollution epidemiology.

Some studies have demonstrated that exposures to particulate matter (aerodynamic diameter ≤2.5 μm, PM_2.5_) and ozone are associated with global oxidative stress [7-11]. Others reported that the exposures were associated with heart rate variability (HRV), plasma homocysteine and C-reactive protein and such effects were modified by genetic polymorphisms related to oxidative defenses [12-16]. In living cells, reactive oxygen species (ROS) are continuously generated as a consequence of metabolic reactions, which may cause oxidative damage to nucleic acids. DNA damage may be repaired by the base excision repair pathway. The resulting repair product, 8-Hydroxy-2'-deoxyguanosine (8-OHdG), is the most common DNA lesion [17] and is not affected directly by either diet or cell turnover [18]. Therefore, 8-OHdG is a good biomarker for ROS or oxidative stress.

A limited number of epidemiological studies reported that 8-OHdG was associated with exposures to indoor and ambient pollution or smoking, but they were conducted among a small number of children or occupationally exposed employees [9,10,19]. Oxidative stress caused by air pollution may be implicated in the development of respiratory disease, cardiovascular disease, lung cancer and other diseases [20-22]. Our recent study found that the elevated urinary 8-OHdG was associated with pollutants often thought of as secondary or formed through photochemical reactions after emission (PM_2.5_, nitrogen dioxide, NO_2_, maximal one-hour ozone, O_3_, sulfate, SO_4_^2-^or organic carbon, OC), but not with directly emitted primary pollutants (black carbon, BC, carbon monoxide, CO or elemental carbon, EC), suggesting that secondary pollution plays a stronger role in oxidative stress [23].

Several studies have demonstrated that certain genetic polymorphisms related to oxidative stress modified effects of PM on cardiovascular responses [6,13,14], but a set of examined single nucleotide polymorphisms (SNPs) was very limited. Further, these studies only indirectly implicated oxidative stress as none of these outcomes was a direct measure of oxidative stress. For example, some studies reported that associations between exposure to PM_2.5 _and heart rate variability (HRV) were modified by polymorphisms of the glutathione-S-transferase M1 (GSTM1) gene [14] or heme oxygenase-1 (HMOX) [15], enzymes that reduce impacts of ROS. Our previous studies examined a set of genotypes related to oxidative stress and found that polymorphisms of hemochromatosis (HFE) and glutathione S-transferase T1 (GSTT1) significantly modified associations of PM_2.5 _with plasma homocysteine [12]. Anh et al. [24] reported that vitamin D-related genes (group-specific component, GC) were significantly associated with the serum D-vitamin concentrations that related to prostate cancer.

However, the selection of certain genes is somewhat arbitrary and the use of an array of genes is vulnerable to false positives from multiple comparisons, a major issue in genetic association studies. In this study, we aimed to examine whether daily ambient OC, SO_4_^2- ^and maximal one-hour O_3 _were associated with urinary 8-OHdG based on our previous findings [23] and such associations were modified by genotypes related to oxidative stress in the Normative Aging Study population (NAS). Because of multiple comparisons, we used the Multiple Testing Procedures (MTP) modified by our team, multtest in the R project http://www.r-project.org to identify significant SNPs from a set of candidate genes [25-28].

## Methods

### Study population

Data were obtained from a longitudinal NAS [29]. Briefly, the NAS is a longitudinal aging population initiated by the Veterans Administration (VA) in 1963. A total of 2,280 men from the greater Boston area free of known chronic medical conditions were enrolled. Subjects were asked to return for examinations every three to five years in the study center, including routine physical examinations, laboratory tests, collection of medical history, social status information, and administration of questionnaires on smoking history, food intake and other factors that may influence health. All participants provided written informed consents and the study protocol was approved by the institutions. By 2006, only did a small proportion of participants remain in the cohort, as many participants had died or were lost to follow up. A total of 320 participants, who still remained in this cohort, were included in our analyses, visiting the clinic between January 2006 and December 2008 for measurement of urinary 8-OHdG and other covariates (no repeated measurements).

### 8-hydroxy-2'-deoxyguanosine and plasma analysis of B vitamins

Urinary 8-OHdG analysis was conducted by Genox Corp (Baltimore, MD). A competitive enzyme-linked immunosorbent assay was used to analyze urinary 8-OHdG [30,31]. The measurement methods have been described elsewhere [23]. Folate, vitamin B6 and B12 in fasting plasma were analyzed at the USDA Human Nutrition Research Center on Aging at Tufts University. Folate and vitamin B12 were examined by radioassay using a commercially available kit from Bio-Rad (Hercules, CA); vitamin B6 (as pyridoxal-5-phosphate) by an enzymatic method using tyrosine decarboxylase. Further details are described elsewhere [32,33]. Plasma creatinine was measured with urine 8-OHdG using spectrophotometric assay. The method has been described elsewhere in details [34].

### Air pollution and Weather Data

Averages of daily OC, SO_4_^2- ^and maximal one-hour O_3 _were used in this study. O_3 _and OC were provided by the Massachusetts Department of Environmental Protection and SO_4_^2- ^was measured at Harvard School Public Health monitoring station. For each day, SO_4_^2-^, OC and O_3 _values were averaged for periods for up to four weeks before the visit as these averaging periods were shown to be most relevant in our previous analyses. Findings from our previous study showed that 8-OHdG were only associated with the secondary pollutants [23]. To adjust for weather condition, we used apparent temperature as an index, defined as a person's perceived air temperature, given the humidity [35].

### Genotypes

In order to avoid the arbitrary selection of genes, we selected all 20 oxidative stress-related SNPs available in the NAS database. We examined effect modification using the array of candidate SNPs, including catalase (CAT, rs480575, rs1001179, rs2284367 and rs2300181), HFE H63 D (rs1799945), HFE C282Y (rs1800562), GSTM1, GSTT1, GSTP1 I105V (rs1695), GSTP1 A114V (rs1799811), HMOX (rs2071746, rs2071747, rs2071749, rs5995098), HMOX-1 VNTR, GC (rs2282679, rs1155563), glutamate cysteine ligase catalytic subunit (GCLC, rs17883901) and glutamate cysteine ligase modifier (GCLM, rs2301022 and rs3170633). HFE is related to cellular uptake of metals that are related to ROS generation and inflammation [8,36]. Glutathione pathways play a vital role in cellular defenses against ROS [14,37-39]. Similarly, GC, GCLC and GCLM are related to glutathione-related metabolism [40,41]. CAT helps catalyze hydrogen peroxide, a powerful ROS into water and molecular oxygen to maintain oxidative balance [39,42]. HMOX-1 was categorized into two levels (any short and both long) based on repeated number of microsatellite (GTn) because previous studies have shown that a high GT repeats at 5'-flanking region may reduce HMOX-1 inducibility by ROS and has been associated with increased risk of cardiovascular diseases [15,43,44]. Previous studies have shown that variations of HFE C282Y, HFE H63 D, HMOX-1, GSTs genes modify associations of PM_2.5 _or BC with HRV or homocysteine [12-15].

Multiplex polymerase chain reaction assays were designed using Sequenom SpectroDESIGNER software (Sequenom Inc, San Diego, Calif) by inputting sequence containing the SNP site and 100 bp of flanking sequence on either side of the SNP. Assays were genotyped using the Sequenom MassArray MALDI-TOF mass spectrometer (Sequonom, CA, USA) with semiautomated primer design (SpectroDESIGNER, Sequenom) and implementation of the very short extension method [45]. Assays failing to multiplex were genotyped using the TaqMan 5' exonuclease [Applied Biosystems (ABI), Foster City, CA, USA] with primers from ABI using radioactive labeled probes detected with ABI PRISM 7900 Sequence Detector System [46].

### Statistical analyses

Statistical analyses were performed with R version 2.9.1. First, we fitted linear regression models to separately examine the association of a single pollutant with urinary 8-OHdG at different day moving averages up to four weeks during the study period to decide which day moving averages for each pollutant were strongly associated with 8-OHdG for effect modification assessment. We used the log-transformation of 8-OHdG to minimize residuals and to stabilize the variance. We identified *a priori *the following variables as important potential confounders based on our previous NAS studies and other studies [9,12,14]: age, body mass index (BMI), alcohol consumption (≥2 drinks/day; yes/no), smoking status (never, former, current), pack-years of cigarettes smoked, plasma folate, vitamin B6, B12, use of statin medication (yes/no) and season and chronic disease status (cardiovascular disease, diabetes and chronic cough). We controlled plasma folate, vitamin B6, B12, age, BMI and pack-years of cigarettes smoked as continuous variables and adjusted for alcohol consumption, smoking status, use of statin medication and season as categorical variables. We adjusted for temperature using three-day moving average of apparent temperature with linear and quadratic terms due to the potential nonlinear relationship between temperature and 8-OHdG. In addition, we adjusted for creatinine clearance rate using the Cockcroft-Gault formula ([140 - age(year)]*weight(kg)]/[72* serum creatinine(mg/dL)]) [47]. We also adjusted for chronic disease status (cardiovascular disease or chronic respiratory diseases) as a dummy variable [23].

We examined effect modification by each of candidate SNP via adding an interaction term of the SNP and the pollutant simultaneously with both the main effect terms adjusting for the same covariates as the above [12,23]. Because two dozens of candidate SNPs were involved in the analyses, results were vulnerable to the multiple comparison problem. To decrease type I errors, we used MTP model to identify the significance of interaction terms of individual SNP and pollutant. The current version of MTP allows one to identify the significance of a group of candidate variables to reduce the false discovery rate meanwhile adjusting for a group of fixed covariates. We used MTP to identify the significance of the group of interaction terms. Because the current version of MTP in R can only include one term that varied across models, our team modified it to include two terms, i.e., the main effect term of genes and the interaction term of one pollutant and genes.

We used the family-wise error rate (fwer) for type I error adjustment, step-down max T (sd.maxT) for method and default values for others in MTP. We briefly described the rationale here. More details about the rationale are described elsewhere [25-27]. MTP is based on Bootstrap estimation of the null distribution samples and the data generating distribution P. Samples are drawn at random with replacement from the observed data. The program generates B bootstrap samples from hypotheses M and obtains M × B samples or M × B matrix of test statistics. Then, based on the M × B matrix of test statistics, the bootstrap estimates or test statistics are induced. There are several methods to define type I error and calculate adjusted p-values in MTP. We selected family-wise error rate and step-down maxT methods in this study. In step-down procedures, the hypotheses corresponding to the most significant test statistics are considered successively, with further tests depending on the outcomes of earlier ones. Therefore, it is more powerful than a single-step. The adjusted p-values for the step-down maxT procedures are given by [26]

$${\tilde{p}}_{rj}=\underset{h=1,\ldots j}{\max}\{\mathrm{Pr}(\underset{l\in \{r_{k},\ldots ,r_{m}\}}{\max}|T_{l}|\geq |t_{rk}||H_{0}^{C})\}$$

where Pr refers to p-value, H denotes hypothesis, and T means test statistic.

MTP directly reported adjusted p-values. An advantage of this method as opposed to only rejection or not of hypotheses, is that it is not needed to determine the level of the test in advance. This study reported adjusted p-values. Then, we quantitatively estimated associations between the pollutants and 8-OHdG across those carrying variants of the significant genes identified by MTP with significant interactions using the bootstrap method with the combination of coefficients of the main effect and the interaction [6].

## Results

Table 1 shows the descriptive statistics of the demographic characteristics, health and environmental variables among the NAS population during 2006-2008 at visit (n = 320). There were no repeated measurements in this study. Table 2 shows distributions of polymorphisms of candidate genes. Among 320 participants, wild types were dominant for CATs, HFEs, GSTP1 (rs1799811), HMOX (rs2071749) and GCLC, but the situation varied for other candidate genes. There were no obvious differences for the distributions of wild and heterozygous types in GCLM, GC and GSTP1 (rs1695). Heterozygous types for HMOX (rs2071746 and rs2071749) consisted of large components. 80.9% and 48.8% of subjects were classified as non-deletions for GSTT1 and GSTM1, respectively. Mean of the HMOX-1 GC repeated number was 25.8 (SD: 3.3) with median 24.

**Table 1:** Descriptive statistics of the demographic characteristics, health and environmental variables among the male Normative Study Aging population at their visits during 2006-2008 at visit (n = 320)

Variable	Values *
Average 8-hydroxy-2'-Deoxyguanosine, ng/ml (log)	2.81 (0.78)

Average maximal 1-hour ozone, ppm	0.039 (0.016)

Average daily sulfate, μg/m^3^	2.68 (2.14)

Average daily organic carbon, μg/m^3^	3.43 (1.31)

Average daily apparent temperature, °C	13.2 (9.8)

Age, years	76.7 (6.1)

Body mass index, kg/m^2^	28.0 (4.5)

Systolic blood pressure, mmHg	124 (18)

Plasma folate, ng/mL	21.6 (12.7)

Plasma pyridoxal-5-phosphate, nmol/L	101 (105.)

Plasma vitamin B_12_, pg/mL	590 (273)

Use of statin, n (%)	180 (56.6)

Cumulative cigarette package years	19.8 (23.4)

Alcohol intake (≥2/day), n (%)	61 (19.4)

Smoking status, n (%)	

Never smoker	93 (29.1)

Current smoker	7 (2.2)

Former smoker	220 (68.8)

* Values are mean ± SD when appropriate. Interquatile ranges (IQR) for 20-day moving averages of maximal 1-hour O_3 _and SO_4_^2- ^were 16.4 ppb and 1.29 μg/m^3^, respectively.

**Table 2:** Genotype distribution of participants (N = 320)*

Polymorphism	Type	Count (%)	Polymorphism	Type	Count (%)
CAT (C/T) rs480575	Wild	138 (49.46)	HFE (G/A) rs1800562	Wild	259 (86.33)

	Heterozygous	113 (40.5)		Heterozygous	41 (13.67)

	Homozygous	28 (10.04)		Homozygous	0 (0)

CAT(A/G) rs1001179	Wild	195 (65.88)	HMOX (A/T) rs2071746	Wild Type	87 (29.49)

	Heterozygous	83 (28.04)		Heterozygous	148 (50.17)

	Homozygous	18 (6.08)		Homozygous	60 (20.34)

CAT(G/A) rs2284367	Wild	160 (55.17)	HMOX (C/G) rs2071747	Wild Type	269 (91.5)

	Heterozygous	109 (37.59)		Heterozygous	25 (8.5)

	Homozygous	21 (7.24)		Homozygous	0 (0)

CAT (A/G) rs2300181	Wild	165 (55.37)	HMOX (G/A) rs2071749	Wild Type	92 (30.77)

	Heterozygous	110 (36.91)		Heterozygous	154 (51.51)

	Homozygous	23 (7.72)		Homozygous	53 (17.73)

GC (C/A) rs2282679	Wild	150 (51.02)	HMOX (C/G) rs5995098	Wild Type	141 (47.32)

	Heterozygous	120 (40.82)		Heterozygous	128 (42.95)

	Homozygous	24 (8.16)		Homozygous	29 (9.73)

GC (T/C) rs1155563	Wild	148 (49.83)	GSTP1 (A/G) rs1695	Wild Type	149 (50.51)

	Heterozygous	128 (43.10)		Heterozygous	123 (41.69)

	Homozygous	21 (7.07)		Homozygous	23 (7.80)

GCLC (C/T) rs17883901	Wild	262 (89.12)	GSTP1 (C/T) rs1799811	Wild Type	254 (86.39)

	Heterozygous	30 (10.20)		Heterozygous	39 (13.27)

	Homozygous	2 (0.68)		Homozygous	1 (0.34)

GCLM (A/G) rs2301022	Wild	116 (39.59)	GSTT1	Deletion	53 (19.13)

	Heterozygous	146 (49.83)		Non deletion	224 (80.87)

	Homozygous	31 (10.58)	GSTM1	Deletion	152 (51.18)

GCLM (A/G) rs3170633	Wild	140 (48.28)		Non deletion	145 (48.82)

	Heterozygous	115 (39.66)	HMOX-1	Both short	21 (6.98)

	Homozygous	35 (12.07)		One short	140 (46.51)

HFE (G/T) rs1799945	Wild	224 (74.17)		Both long	140 (46.51)

	Heterozygous	71 (23.51)			

	Homozygous	7 (2.32)			

*The sum of the subjects in each genotype may not add up to the total number of subjects due to missing genotyping data. Missing genotyping is due to a variable number of samples for each locus for which genotyping was not successful.

We first fit the linear regression model to estimate associations of OC, SO_4_^2- ^and maximal one-hour O_3 _with 8-OHdG using moving averages of pollutants up to four weeks. Results show that main effects varied across different day moving averages and 24-, 20- and 18-day moving averages were strongest associated with SO_4_^2-^, OC and maximal one-hour O_3_, respectively, which were used to assess effect modifications. The detailed information has been reported elsewhere [23]. For an IQR increases in 24-, 20- and 18-day moving averages of daily SO_4_^2-^, OC and maximal one-hour O_3_, urinary 8-OHdG increased by 29.0% (95% CI: 5.9%, 52.1%), 27.6% (95% CI: 3.6%, 51.6%) and 54.3% (95% CI: 7.6%, 100.9%), respectively.

**Figure 1:**
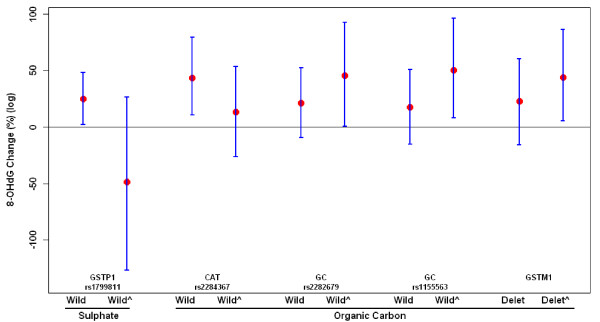
**Estimated percent changes in 8-OHdG (log) (95% confident interval) associated with a unit increase of 17- and 20-day moving averages of organic carbon and sulfate, respectively by gene polymorphisms**. Adjusting for apparent temperature, age, body mass index, smoking status, pack-years of cigarettes smoked, alcohol consumption, use of statin medication, plasma folate, vitamin B6 and B12, season, chronic disease and creatinine clearance rate. Wild^: non-wild; Delet: deletion, delet^: non-deletion.

Before examining effect modification, we categorized each candidate gene into a dummy variable so that the gene and the pollutant of interest only have one interaction term. We combined the homozygous and heterozygous types for appropriate genes known as the non-wild type (dominant model) due to small number of the homozygous type. We also combined the homozygous and heterozygous short repeat for HMOX-1, referred to as any short (Table 2). Then, we identified candidate genes that executed significant effect modification as aforementioned. Adjusted p-values in MTP model show that GSTP1 A114V (rs1799811) marginally significantly modified the effect of SO_4_^2- ^on 8-OHdG (adjusted *p *= 0.091). CAT (rs2286367) (adjusted p = 0.037), GSTM1 (adjusted p = 0.037), GC (rs2282679) (adjusted *p *= 0.025) and GC (rs1155563) (adjusted p = 0.027) significantly modified effects of OC on 8-OHdG. There was no significant effect modification for O_3 _(Table 3). As sensitive analyses, we used different options in MTP for typeone (type I error) (tail probabilities for error rate, TPPER; false discovery rate, FDR) and *methods *(single-step maximum T, ss.maxT; single-step minimum P ss.minP; step-down minimum P, ss.minP). Similar trends were found in spite of some variations. We also categorized pack-years of cigarettes smoked using tertiles as cut-off and re-ran MTP model. Results were similar to those using continuous variable for pack-years of cigarettes smoked. Figure 1 shows the estimated effects of OC or SO_4_^2- ^on 8-OHdG across subpopulations carrying different genotypes, for those SNPs where an interaction with p < 0.10 was found.

**Table 3:** Statistical p-values for the interaction between pollutants and SNPs from MTP model using family-wise error rate and step-down max T method *

SNP	OC	**SO**_**4**_^**2-**^	**O**_**3**_
CAT (C/T) rs480575	0.770	1.000	1.000

CAT(A/G) rs1001179	0.770	0.825	0.749

CAT(G/A) rs2284367	0.037	0.771	0.531

CAT (A/G) rs2300181	0.131	0.976	1.00

GC (C/A) rs2282679	0.025	1.000	0.999

GC (T/C) rs1155563	0.027	1.000	0.999

GCLC (C/T) rs17883901	0.896	1.000	0.999

GCLM (A/G) rs2301022	0.745	1.000	1.000

GCLM (A/G) rs3170633	0.368	0.995	1.000

HFE (G/T) rs1799945	0.997	0.995	1.000

HFE (G/A) rs1800562	0.417	1.000	1.000

HMOX (A/T) rs2071746	0.368	0.995	1.000

HMOX (C/G) rs2071747	0.177	0.732	0.999

HMOX (G/A) rs2071749	0.770	1.000	1.000

HMOX (C/G) rs5995098	0.177	1.000	1.000

GSTP1 (A/G) rs1695	0.997	0.995	1.000

GSTP1 (C/T) rs1799811	0.997	0.091	0.994

GSTT1	0.177	0.965	1.000

GSTM1	0.037	0.984	1.000

HMOX-1	0.758	1.000	1.000

* using 24-, 20- and 18-day moving averages of OC, SO_4_^2- ^and maximal 1-hour O_3_, respectively.

## Discussion

We found that associations of the secondary pollutants, specifically OC and SO_4_^2-, ^with 8-OHdG, a direct oxidative stress-related biomarker, were modified by polymorphisms in genes related to oxidative defenses. This is significant for several reasons. First, the finding that genetic polymorphisms in the oxidative defense pathway modified the association suggests that it is not due to chance or confounding, since neither should be associated with the genotypes of the individuals. Second, while considerable focus has been placed recently on freshly generated traffic particles, such as BC or ultrafine particle number, this study confirms that particles, including particles from coal burning power plants, play a role in increasing systemic oxidative stress.

The specific polymorphisms that modified the associations were GSTP1 (rs1799811), GSTM1, CAT (rs1799811) and GC (rs22826799, rs1155563). We found 8-OHdG was more strongly associated with SO_4_^2- ^among those carrying the wild type of the GSPT1, and more strongly associated with OC among those carrying the wild type of CAT (rs2284367), the non-deletion of GSTM1 and the non-wild type of the GCs (rs2282679 and rs1155563) comparing with other types of the corresponding genes (Figure 1). Based on our knowledge, it is the first time that MTP has been used to identify significant gene-environment interactions. MTP has advantages over some other approaches to controlling for false discovery rates in which a group of fixed covariates are adjusted for while a set of variables were compared.

Several studies have examined effect modification and found that people carrying variants of oxidative stress-related genes are differentially susceptible to air [12-14,16,48]. Human GSTs are subdivided into several classes, among which GSTT1, GSTM1 and GSTP1 have been extensively investigated [12,14,49,50]. GSTM1 or GSTT1 catalyzes the conjugation of glutathione to numerous potentially genotoxic compounds [50]. Individuals with the deletion of GSTM1 or GSTT1 have been shown to reduce GST activity and thus may be unable to eliminate toxins as efficiently when they expose to oxidative pollutants [50]. Schwartz et al. [14] found that PM_2.5 _was significantly associated with high frequency of HRV among those without the GSTM1 allele, but not for those with the allele. Gilliland et al. [48] reported that exposure to *in utero *maternal smoking was associated with increased prevalence of early onset asthma among those without GSTM1 allele, but not for those with GTSM1 allele. Similarly, Romieu et al. [51] found that GSTM1 null children were more sensitive to ozone exposure. However, all the aforementioned studies did not report whether there were significant effect modifications. Differential results from these stratification analyses might also be attributed to statistical powers across subpopulations or differential distributions of other controlled or uncontrolled covariates across subpopulations. This study observed that GSTM1 significantly modified associations of OC with 8-OHdG, but paradoxically that the GSTM1 null allele provided protection against exposure. Our recent study examined whether variations of a set of genes altered effects of black carbon and PM_2.5 _on plasma homocysteine in this population and found that GSTT1 (but not GSTM1) significantly modified associations between pollutants and homocysteine. PM_2.5 _and black carbon were more strongly associated with homocysteine among those carrying GSTM1 allele comparing those without the allele although no significant interactive effects were found [12]. Different findings of effect modification by GSTM1 variation across studies may reflect differences of exposure, outcome and population, measurement errors in exposure or phenotype, and by chance. Similar situations also appeared in other studies [52,53]. Therefore, statistical effect modification may be inconsistent with biological interaction. Further research or meta-analysis is needed for GSTM1.

In contrast, few studies have examined the function of GSTP1 A114V (rs1799811) on diseases with inconsistent results [54-57]. None of these studies found the GSTP1 is significantly associated with the outcomes of interest although some studies found positive trends. Therefore, the functions of the polymorphisms have not been determined. Several studies examined effect modifications of GSTT1 on various endpoints but no significant effect modification was found [58-60]. For example, Melén et al. [59] examined whether GST modified traffic-related pollution effect on childhood allergic disease and found that carriers with variants of GSTP1 (rs1799811) were higher susceptible to NO_x_. Our study found the variation of GSTP1 showed a protective effect of SO_4_^2- ^on 8-OHdG. However, other two studies did not find any evidence that the GSTP1 modified effects of black carbon or smoking on blood pressure or Parkinson's disease occurrence [58,60]. Inconsistent observed findings may be attributable to various sources as aforementioned. In this study, it may also related to the small number of variants in this population, which probably lead to unstable estimates. Therefore, its functions remain to be clarified by others (Table 2).

GC, vitamin D-related genes, is related to the vitamin D metabolism [61]. Vitamin D is activated to form 1, 25-dihydroxyvitamin D in the liver and kidney and then transported in serum to different tissues by the vitamin D-binding protein, which is encoded by GC [61]. Studies show that polymorphisms of vitamin D-related genes are associated with various cancers, cardiovascular diseases and respiratory diseases [62-64]. Ahn et al. [61] examined variations of 212 SNPs related to vitamin D metabolism and found that all four SNPs of *GC *(rs1212631, rs2282679, rs7041, rs1155563) are significantly associated with the concentration of serum vitamin D. When these four SNPs were simultaneously included in the multivariate model, only two SNPs (rs22679, rs1155563) were significantly associated with vitamin D. In this study, we found that the two SNPs of GC (rs22679, rs1155563) were associated with 8-OHdG in this study. The mechanisms remain to be clarified yet.

Catalase is a protein of 526 amino acids, encoded by the catalase gene with 34 kb pairs of nuclear acids [65]. Catalase is the main regulator of hydrogen peroxide metabolism [66]. Catalase enzyme mutations may reduce its activity and probably results in the increase of the hydrogen peroxide concentrations in the tissues [62]. Inherited catalase deficiency results in acatalasemia (homozygous state) and hypocatalasemia (heterozygous) and is related to increased plasma homocysteine concentrations [42,67,68]. Our previous study reported that the variation of CAT modified associations between particle matter and plasma homocysteine concentrations [12].

Experimental toxicology studies have shown that air pollutants act via the oxidative stress pathway [8,36,69]. Ghio et al. [36] found that homozygous Belgrade rats functionally deficient in divalent metal transporter-1 display decreased metal transport from the lower respiratory tract and have stronger lung injury than control littermates, when exposed to oil fly ash containing iron. Belgrade rats cannot transport iron and other divalent metals across membranes via HFE gene regulated processes. They also reported that healthy volunteers exposed to concentrated ambient air particles had increased concentrations of blood fibrinogen and induced mild pulmonary inflammation [8]. Tamagawa et al. [69] reported that five-day and four-week exposures to PM_10 _caused acute and chronic lung and systematic inflammation of New Zealand rabbits.

There are several strengths in this study. First, we used MTP model to identify the significance of a group of candidate genes while we examined effect modification by genes on air pollution effects. This method overcame some problems in this kind of studies, such as arbitrary selection of a few significant genes or high false discovery rate when individually examining a set of genes. Secondly, this study was conducted in a relatively large population. Information of participants was well measured and collected. However, several limitations also exist with this study. First, we used air pollution data collected from a single monitoring site for personal pollution exposure and therefore, some extent misclassification might happen. A recent study compared ambient concentrations with personal exposures with monitoring measurement and results show that ambient measures were good surrogates for PM_2.5 _and SO_4_^2- ^in both winter and summer, but O_3 _was only good in summer, not well in winter [70]. Nevertheless, with non-differential misclassification, any potential bias would be expected toward the null. Second, MTP has several options to select type I error and several methods to calculate adjusted p-values. Using bootstrap re-sampling methods will result in different estimates when a MTP model is rerun. These will introduce the uncertainties in model selections [25-28]. In addition, the NAS consists of an aged population and non-Hispanic white men were dominant. Thus, the findings are not well generalizable to other populations.

## Conclusions

This study found that variations of oxidative stress-related genes modified effects of OC or SO_4_^2- ^on 8-OHdG. This suggests that effects of OC or SO_4_^2- ^on 8-OHdG and other endpoints may be through the oxidative stress pathway.

## Abbreviations

BC: black carbon; OC: organic carbon; EC: element of carbon; SNP: single nucleotide polymorphism; NO2: nitrogen dioxide; CO: carbon monoxide; O3: ozone; 8-OHdG: 8'-hydroxy-2'-deoxyguanosine; PM_2.5_: particulate matter ≤2.5 μm in aerodynamic diameter; GST: glutathione S-tranferase; CAT: catalase; GC: group-specific component; HFE: hemochromatosis; HOMX: heme oxygenase-1; GCLC: glutamate cysteine ligase catalytic subunit; GCLM: glutamate cysteine ligase modifier;

## Competing interests

The authors declare that they have no competing interests.

## Authors' contributions

CR was responsible for study design, data analyses, result interpretation and manuscript writing. JS was responsible for study design, data collection and result interpretation. Other coauthors participated in the study design, data collection and result interpretation. All authors read and approved the final manuscripts.

## References

[B1] Schwartz J The effects of particulate air pollution on daily deaths: a multi-city case-crossover analysis Occup Environ Med 2004 61 956 961 10.1136/oem.2003.008250 15550600 PMC1740692

[B2] Bell ML McDermott A Zeger SL Samet JM Dominici F Ozone and short-term mortality in 95 US urban communities, 1987-2000 JAMA 2004 292 2372 2378 10.1001/jama.292.19.2372 15547165 PMC3546819

[B3] Dominici F Peng RD Bell ML Pham L McDermott A Zeger SL Samet JM Fine particulate air pollution and hospital admission for cardiovascular and respiratory diseases JAMA 2006 295 1127 1134 10.1001/jama.295.10.1127 16522832 PMC3543154

[B4] Zanobetti A Schwartz J Particulate air pollution, progression, survival after myocardial infarction Environ Health Perspect 2007 115 769 775 10.1289/ehp.9201 17520066 PMC1867961

[B5] Ren C Willims GM Morawska L Mengersen K Tong S Ozone modifies associations between temperature and cardiovascular mortality: analysis the NMMAPS data Occup Environ Med 2008 65 255 260 10.1136/oem.2007.033878 17890300

[B6] Ren C Baccarelli A Wilker E Suh H Sparrow D Vokonas P Wright R Schwartz J Lipid and endothelial related genes, ambient particulate matter, and heart rate variability --the VA Normative Aging Study J Epidemiol Community Health 2010 64 49 56 10.1136/jech.2008.083295 19602472 PMC3935361

[B7] Brook RD Cardiovascular effects of air pollution Clin Sci 2008 115 175 187 10.1042/CS20070444 18691154

[B8] Ghio AJ Kim C Devlin RB Concentrated ambient air particles induce mild pulmonary inflammation in healthy human volunteers Am J Respir Crit Care Med 2000 162 981 988 10988117 10.1164/ajrccm.162.3.9911115

[B9] Kim JY Mukherjee S Ngo L Christiani DC Urinary 8-hydroxy-2'-deoxyguanosine as a biomarker of oxidative DNA damage in workers exposure to fine particles Environ Health Perspect 2004 112 666 671 10.1289/ehp.6827 15121508 PMC1241959

[B10] Gurgueira SA Lawrence J Coull B Murthy GK González-Flecha B Rapid increases in the steady-state concentration of reactive oxygen species in the lungs and heart after particulate air pollution inhalation Environ Health Perspect 2002 110 749 755 10.1289/ehp.02110749 12153754 PMC1240944

[B11] Vinzents PS Møller P Sørensen M Knudsen LE Hertel O Jensen FP Schibye B Loft S Personal exposure to ultrafine particles and oxidative DNA damage Environ Health Perspect 2005 113 1485 1490 10.1289/ehp.7562 16263500 PMC1310907

[B12] Ren C Park SK Vokonas PS Sparrow D Wilker E Baccarelli A Suh H Schwartz J Air pollution and homocysteine: more evidence that oxidative stress-related genes modify effects of particulate air pollution Epidemiology 2010 21 198 206 10.1097/EDE.0b013e3181cc8bfc 20110814 PMC3939788

[B13] Park SK O'Neill MS Wright RO Hu H Vokonas PS Sparrow D Suh H Schwartz J HFE genotype, particulate air pollution, and heart rate variability--a gene-environment interaction Circulation 2006 114 2798 2805 10.1161/CIRCULATIONAHA.106.643197 17145987

[B14] Schwartz J Park SK O'Neill MS Vokonas PS Sparrow D Welss S Kelsey K Glutathione-S-transferase M1, obesity, statins, and autonomic effects of particles: gene-by-drug-by-environment interaction Am J Respir Crit Care Med 2005 172 1529 1533 10.1164/rccm.200412-1698OC 16020798 PMC2718454

[B15] Chahine T Baccarelli A Litonjua A Write RO Suh H Gold DR Sparrow D Vokonas P Schwartz J Particulate air pollution, oxidative stress genes, and heart rate variability in an elderly cohort Environ Health Perspect 2007 115 1617 1622 10.1289/ehp.10318 18007994 PMC2072834

[B16] Zeka A Sullivan JR Vokonas PS Sparrow D Schwartz J Inflammatory markers and particulate air pollution: characterizing the pathway to disease Int J Epidemiol 2006 35 1347 1354 10.1093/ije/dyl132 16844771

[B17] Kasai H Crain PF Kuchino Y Nishimura S Ootsuyama A Tanooka H Formation of 8-hydroxygunine moiety in cellular DNA by agents producing oxygen radicals and evidence for its repair Carcinogenesis 1986 7 1849 1851 10.1093/carcin/7.11.1849 3769133

[B18] Cooke MS Evans MD Dove R Rozalski R Gackowski D Siomek A Lunec J Olinski R DNA repair is responsible for the presence of oxidative damaged DNA lesions in urine Mutat Res 2005 574 1-2 58 66 15914207 10.1016/j.mrfmmm.2005.01.022

[B19] Lu CY Ma YC Lin JM Chuang CY Sung FC Oxidative DNA damage estimated by urinary 8-hydroxydeoxyguanosine and indoor air pollution among non-smoking office employees Environ Res 2007 103 331 337 10.1016/j.envres.2006.08.009 17034784

[B20] Chuang KJ Chang CC Su TC Lee CT Tang CS The effect of urban air pollution on inflammation, oxidative stress, coagulation, and autonomic dysfunction in young adults Am J Respir Crit Care Med 2007 176 370 376 10.1164/rccm.200611-1627OC 17463411

[B21] Wiseman H Halliwell B Damage to DNA by reactive oxygen and nitrogen species: role in inflammatory disease and progression to cancer Biochem J 1996 313 17 29 8546679 10.1042/bj3130017 PMC1216878

[B22] Higashi Y Noma K Yoshizumi M Kihara Y Endothelial function and oxidative stress in cardiovascular diseases Circ J 2009 73 411 418 10.1253/circj.CJ-08-1102 19194043

[B23] Ren C Fang S Wright RO Suh H Schwartz J Urinary 8-hydroxy-2'-deoxyguanosine as a biomarker of oxidative DNA damage induced by ambient pollution in the Normative Aging Study Occup Environ Med 2010 [Online Oct 27, 2010] 20980452 10.1136/oem.2010.056358 PMC3786183

[B24] Anh J Albanes D Berndt SI Peters U Chatterjee N Freedman ND Abnet CC Huang WY Kibel AS Crawford DE Weinstein SJ Chanock SJ Schatzki A Hayes RB Vitamin D-related genes, serum vitamin D concentrations and prostate cancer risk Carcinogenesis 2009 30 5 769 776 10.1093/carcin/bgp055 19255064 PMC2675652

[B25] Pollard KS Dudiot S van der Lann MJ Multiple testing procedures: R multtest package and application to genetics 2005 http://www.bepress.com/ucbbiostat/paper164/15693941

[B26] Dudoit S Shaffer JP Boldrick JC Multiple hypothesis testing in microarray experiments Stat Sci 2003 18 71 103 10.1214/ss/1056397487

[B27] Dudoit S van der Laan M Pollard KS Multiple testing part I: single-step procedures for control of general type I error rates Stat Appl Genet Mol Biol 2004 3 13 10.2202/1544-6115.1040 16646791

[B28] van der Laan M Dudoit S Pollard KS Multiple testing part II: step down procedures for control of family-wise error rate Stat Appl Genet Mol Biol 2004 3 14 10.2202/1544-6115.1041 16646792

[B29] Bell B Rose C Damon A The veterans Administration longitudinal study of healthy aging Gerontologist 1966 6 179 184 5342911 10.1093/geront/6.4.179

[B30] Erhola M Toyokuni S Okada K Tanaka T Hiai H Ochi H Uchida K Osawa T Nieminen MM Alho H Kellokumpu-Lehtinen Pl Biomarker evidence of DNA oxidation in lung cancer patients: association of urinary 8-hydroxy-2'-deoxyguanosine excretion with radiotherapy, chemotherapy, and response to treatment FEBS lett 1997 409 287 291 10.1016/S0014-5793(97)00523-1 9202163

[B31] Leinonen J Lehtimaki T Toyokuni S Okada K Tanaka T Hiai H Ochi H Laippala P Rantalaiho V Virta O Pasternack A Alho H New biomarker evidence of oxidative DNA damage in patients with non-insulin-dependent diabetes mellitus FEBS Lett 1997 417 150 152 10.1016/S0014-5793(97)01273-8 9395094

[B32] Park SK O'Neill MS Vokonas PS Sparrow D Spiro A III Tucker KL Suh H Hu H Schwartz J Traffic-related particles are associated with elevated homocysteine - the VA Normative Aging Study Am J Respir Crit Care Med 2008 178 283 289 10.1164/rccm.200708-1286OC 18467508 PMC2542426

[B33] Tucker KL Qiao N Scott T Rosenberg I Spiro A III High homocysteine and low B vitamins predict cognitive decline in aging men: the Veterans Affairs Normative Aging Study Am J Clin Nutr 2005 82 627 635 16155277 10.1093/ajcn.82.3.627

[B34] Bowers L Wong E Kinetic serum creatinine assays. II. A critcal evaluation and review Clin Chem 1980 26 555 7020989

[B35] Kalkstein L Valamont K An evaluation of summer discomfort in the United States using a relative climatologic index Bull Am Meteorol Soc 1986 67 842 848 10.1175/1520-0477(1986)067<0842:AEOSDI>2.0.CO;2

[B36] Ghio AJ Piantadosi CA Wang X Divalent metal transporter-1 decreases metal-related injury in the lung Am J Physiol Lung Cell Mol Physiol 2005 289 460 467 10.1152/ajplung.00154.2005 15908475

[B37] Hayes JD McLellan LI Glutathione and glutathione dependent enzymes respresent a co-ordinately regulated defense against oxidative stress Free Radic Res 1999 31 273 300 10.1080/10715769900300851 10517533

[B38] Gilliland FD LI YF Saxon A Diaz-Sanchez D Effect of glutathione-S-transferase M1 and P1 genotypes on xenobiotic enhancement of allergic responses: randomized, placebo-controlled crossover study Lancet 2004 363 119 125 10.1016/S0140-6736(03)15262-2 14726165

[B39] Forsberg L Lyrenäs L de Faire U Morgenstern R A common functional C-T substitution polymorphisms in the promoter region of the human catalase gene influences transcription factor binding, reporter gene transcription and is correlated to blood catalase levels Free Radic Biol Med 2001 30 500 505 10.1016/S0891-5849(00)00487-1 11182520

[B40] Engstöm KS Strömberg U Lundh T Johansson I Vessby B Hallmans G Skerfving S Broberg K Genetic variation in glutathione-related genes and body burden of methylmercury Environ Health Perspect 2008 116 734 739 18560528 10.1289/ehp.10804 PMC2430228

[B41] Siedlinski M Postma DS van Diemen CC Blokstra A Smit HA Boezen HM Lung function loss, smoking, vitamin C intake, and polymorphisms of the glutamate-cysteine ligase genes Am J Respir Crit Care Med 2008 178 13 19 10.1164/rccm.200711-1749OC 18420959

[B42] Góth L Vitai M The effects of hydrogen peroxide promoted by homocysteine and inherited catalase deficiency on human hypocatalasemic patients Free Radic Biol Med 2003 35 882 888 14556852 10.1016/s0891-5849(03)00435-0

[B43] Chen YH Lin SJ Lin MW Tsai HL Kuo SS chen JW Charng MJ Wu TC Chen LC Ding PYA Pan WH Jou YS Chau LY Microsatellite polymorphism in promoter of heme oxygenase-1 gene is associated with susceptibility to coronary artery disease in type 2 diabetes patients Hum Genet 2002 111 1 8 10.1007/s00439-002-0769-4 12136229

[B44] Kaneda H Ohno M Taguchi J Togo M Hashimoto H Ogasawara K Aizawa T Ishizaka N Nagai R Heme oxygenase-1 gene promoter polymorphism is associated with coronary artery disease in Japanese patients with coronary risk factors Arterioscler Thromb Vasc Biol 2002 22 1680 1685 10.1161/01.ATV.0000033515.96747.6F 12377749

[B45] Sun X Ding H Hung K Guo B A new MALDI-TOF based mini-sequencing assay for genotyping of SNPs Nucleic Acids Res 2000 28 e68 10.1093/nar/28.12.e68 10871391 PMC102753

[B46] Lee LG Connell CR Bloch W Allelic discrimination by nick-translation PCR with fluorogenic probes Nucleic Acids Res 1993 21 3761 3766 10.1093/nar/21.16.3761 8367293 PMC309885

[B47] Cockcroft DW Gault MH Prediction of creatinine clearance from serum creatinine Nephron 1976 16 31 41 10.1159/000180580 1244564

[B48] Gilliland FD Li Y Dubeau L Berhane K Avol E Gauderman WJ Peters JM Effects of glutathione-S-transferase M1, maternal smoking during pregnancy, and environmental tobacco smoke on asthma and wheezing in children Am J Respir Crit Care Med 2002 166 457 463 10.1164/rccm.2112064 12186820

[B49] Bergamaschi E De Palma G Mozzoni P Vanni S Vettori MV Broeckaert F Bernard A Mutti A Polymorphism of quinone-metabolizing enzymes and susceptibility to ozone-induced acute effects Am J Respir Crit Care Med 2001 163 1426 1431 11371413 10.1164/ajrccm.163.6.2006056

[B50] Couphlin SS Hall IJ Glutahione S-transferase polymorphisms and risk of ovarian cancer: a HuGE review Genet Med 2002 4 250 257 10.1097/00125817-200207000-00003 12172391

[B51] Romieu I Sienra-Monge JJ Ramírez-Aguilar M Moreno-Macías H Reyes-Ruiz NI Estela del Rio-Navarro B Hernández-Avila M London SJ Genetic polymorphism of GSTM1 and antioxidante supplementation influence lung function in relation to ozone exposure in asthmatic children in Mexico City Thorax 2004 59 8 10 14694237 PMC1758856

[B52] Gilliland FD Rappaport EB Berhane K Islam T Dubeau L Gauderman WJ McConnell R Effects of glutathione S-transferase P1, M1, and T1 on acute respiratory illness in school children Am J Respir Crit Care Med 2002 166 346 351 10.1164/rccm.2111048 12153968

[B53] Gilliland FD Gauderman WJ Vora H Rappaport E Dubeau L Effects of glutathione-S-transferase M1, T, and P1 on childhood lung function growth Am J Respir Crit Care Med 2002 166 710 716 10.1164/rccm.2112065 12204870

[B54] Al-Dyyel F Al-Rasheed M Ibrahim M Bu R Bavi P Abubaker J Al-Jomah N Mohamed GH Moorji A Uddin S Siral AK Al-Kuraya K Polymorphisms of drug-metabolizing enzymes CYP1A1, GSTT and GSTP contributed to the development of diffuse large B-cell lymphoma risk the Saudi Arabian population Leuk Lymphoma 2008 49 122 129 10.1080/10428190701704605 18203021

[B55] Gemignani F Landi S Szeszenia-Dabrowska N Zaridze D Lissowska J Rudnai P Fabianova E Mates D Foretova L Janout V Bencko V Gaborieau V Gioia-Patricola L Bellini1 I Barale R Canzian F Hall J Boffetta P Hung RJ Brennan P Development of lung cancer before the age of 50: the role of xenobiotic metabolizing genes Carcinogenesis 2007 28 1287 1293 10.1093/carcin/bgm021 17259654

[B56] Yang XR Pfeiffer PM Goldstein AM Influence of glutathione-S-transferase (GSTM1, GSTP1, GSTT1) and cytochrome p450 (CYP1A, CYP2D6) polymorphisms on numbers of basal cell carcinomas (BCCs) in families with the naevoid basal cell carcinoma syndrome J Med Genet 2006 43 e16 10.1136/jmg.2005.035006 16582078 PMC2563218

[B57] De Roos AJ Gold LS Wang S Hartge P Cerhan JR Cozen W Yeager M Chanock S Rothman N Severson RK Metabolic gene variants and risk of non-Hodgkin's lymphoma Cancer Epidemiol Biomarkers Prev 2006 15 1647 1653 10.1158/1055-9965.EPI-06-0193 16985026

[B58] Wahner AD Glatt CE Bronstein JM Ritz B Glutathione S-transferase mu, omega, pi, and theta class variants and smoking in Parkinson's disease Neurosci Lett 2007 413 274 278 10.1016/j.neulet.2006.11.053 17194543 PMC1864949

[B59] Melén E Nyberg F Lindgren CM Berglind N Zucchelli M Nording E Hallberg J Svartengren M Morgenstern R Kere J Bellander T Wickman M Pershagen G Interactions between glutathione S-transferase P1, tumor necrosis factor, and traffic-related air pollution for development of childhood allergic disease Environ Health Perspect 2008 116 1077 1084 18709160 10.1289/ehp.11117 PMC2516580

[B60] Mordukhovich I Wilker E Suh H Wright R Sparrow D Vokonas PS Schwartz Black carbon exposure, oxidative stress genes, and blood pressure in a repeated measures study Environ Health Perspect 2009 117 1767 1772 20049130 10.1289/ehp.0900591 PMC2801196

[B61] Ahn J Albanes D Berndt SI Peters U Chatterjee N Freedman ND Abnet CC Huang W Kibel AS Crawford ED Weinstein SJ Chanock SJ Schatzikin A Hayes RB Vitamin D-related genes, serum vitamin D concentrations and prostate cancer risk Carcinogenesis 2009 30 769 776 10.1093/carcin/bgp055 19255064 PMC2675652

[B62] Raimondi S Johansson H Maisonneuve P Gandini S Review and meta-analysis on vitamin D receptor polymorphisms and cancer risk Carcinogenesis 2009 30 1170 1180 10.1093/carcin/bgp103 19403841

[B63] McCullough ML Bostick RM Mayo TL Vitamin D gene pathway polymorphisms and risk of colorectal, breast, and prostate cancer Annu Rev Nutr 2009 29 111 132 10.1146/annurev-nutr-080508-141248 19400699

[B64] Wang TJ Pencina MJ Booth SL Jacques PF Ingelsson E Lanier K Benjamin EJ D'Agostino RB Wolf M Vasan RS Vitamin D deficiency and risk of cardiovascular disease Circulation 2008 117 503 511 10.1161/CIRCULATIONAHA.107.706127 18180395 PMC2726624

[B65] Quan F Korneluk RG Tropak MB Gravel RA Isolation and characterization of the human catalase gene Nucleic Acids Res 1986 14 5321 5335 10.1093/nar/14.13.5321 3755525 PMC311543

[B66] Mueller S Riedel HD Stremmel W Direct evidence for catalase as the predominant H_2_O_2 _removing enzyme in erythrocytes Blood 1997 90 4973 4978 9389716

[B67] Ahn J Nowell S McCann SE Yu J Carter L Lang NP Kadlubar FF Ratnasinghe LD Ambrosone CB Associations between catalase phenotype and genetype: modification by epidemiologic factors Cancer Epidemiol Biomarkers Prev 2006 15 1217 1222 10.1158/1055-9965.EPI-06-0104 16775184

[B68] Góth L Rass P Páy A Catalase enzyme mutations and their association with diseases Mol Diagn 2004 8 141 149 15771551 10.1007/BF03260057

[B69] Tamagawa E Bai N Morimoto K Yatera E Zhang X Xing L Li Y Laher I Sin DD Man SFP van Eeden SF Particulate matter exposure induces persistent lung inflammation and endothelial dysfunction Am J Physiol Cell Mol Physiol 2008 295 79 85 10.1152/ajplung.00048.2007 PMC2494798 18469117

[B70] Sarnat JA Brown KW Schwartz J Coull BA Koutrakis P Ambient gas concentrations and personal particulate matter exposures: implications for studying the health effects of particles Epidemiology 2005 16 385 395 10.1097/01.ede.0000155505.04775.33 15824556

